# Recognition of Human Gait Under Asymmetric Loading

**DOI:** 10.3390/s26061940

**Published:** 2026-03-19

**Authors:** Marcin Derlatka

**Affiliations:** Institute of Biomedical Engineering, Faculty of Mechanical Engineering, Bialystok University of Technology, 15-351 Bialystok, Poland; m.derlatka@pb.edu.pl; Tel.: +48-571-443-044

**Keywords:** biometrics, human gait recognition, ground reaction forces, ensemble classifiers, classification, asymmetric load

## Abstract

Biometric recognition of human gait is a promising, non-invasive method for the identification of people that does not require their engagement. Existing solutions mainly focus on the identification effectiveness under laboratory conditions, frequently overlooking factors that disrupt the gait of test subjects. The present work considers the issue of identifying a person on the basis of ground reaction forces in cases where their gait is disrupted through asymmetric loading. This paper proposes a solution based on ensemble classifiers utilizing various types of deep neural networks as base classifiers. To further increase the ability to generalize base models, data augmentation was used. The proposed solution was tested on a sample of 215 people (7351 gait cycles) and two strategies for combining classifier decisions. The accuracy results obtained, ranging between 99.8, 98.55, and 98.85% correct recognitions depending on the scenario analyzed, are very good and significantly exceed other methods presented in the literature to date.

## 1. Introduction

The recognition of people on the basis of their gait is an example of behavioral biometrics where identification is based on dynamic movement patterns resulting from personal qualities of the neuromuscular system and biomechanics of locomotion [1,2]. In contrast to physiological biometrics, such as fingerprints [3], faces [4], or irises [5], human gait is a dynamic process that is the result of a complex synergy between the nervous, muscular, and skeletal systems. It is also unique for every person and relatively constant through time [6]. An important quality of behavioral biometrics, which makes it more difficult to apply on a large scale for the identification of people, is its greater susceptibility to the impact of internal and external factors than physical biometrics. For that reason, gait recognition systems should be assessed not only on their effectiveness under laboratory conditions but, primarily, on their resilience to covariates and their ability to generalize when faced with conditions not present during the training phase.

In the case of gait biometrics, covariate factors that affect recognition quality arise from the measurement method or are independent of it and stem from factors influencing the way a person moves. Among the latter, the most mentioned factors include walking speed [7], footwear [8], walking surface [9], or diseases [10]. Concerning the measurement method, three main groups of signals can be distinguished:

- Camera-based systems;
- Wearable devices such as gyroscopes or accelerometers;
- Force plates and pressure mats.

Systems based on information obtained using video cameras are most utilized in gait recognition research. There are large, publicly available databases, such as CASIA-B and OU-ISIR, that enable the development and comparison of recognition algorithms [11,12,13]. Biometric systems based on video signals are particularly susceptible to factors such as changes in viewing angle; camera distance; contrast and brightness conditions; image resolution; and the obstruction of the silhouette by clothing, carried objects, other people, or elements of the surrounding infrastructure [7,14].

Methods utilizing wearable devices, particularly inertial sensors built into smartphones or dedicated IMU systems, are robust to lighting and viewing conditions but require the sensor to be physically worn by the user, which limits their application in demanding, fully non-invasive, contactless, or covert monitoring. Systems of this type are sensitive to the position of the sensor, as well as other operational parameters such as sampling frequency [15,16]. It has also been noted that although a greater number of sensors increases accuracy [17], it invariably reduces user comfort, which, indirectly, affects the way a person moves [16].

Systems utilizing the measurement of ground reaction forces (GRFs) and plantar pressure distributions represent a particularly interesting, although still relatively less explored, modality. In contrast to biometric systems that use video and inertial sensors, GRF measurements provide information directly related to movement dynamics and postural control mechanisms. The work of Pataky et al. [18] demonstrated that spatiotemporal plantar pressure distributions are highly unique to each individual, even within large populations, making them useful as a potential biometric trait. Systems of this kind record signals whose values depend, among other factors, on the body mass of the subject, thus making them susceptible to situations where it is altered as a result, for example, of carrying items such as a briefcase or a backpack. It should be noted that GRFs, on the one hand, remain correlated with a person’s body mass, which, in practice, makes identity recognition noticeably easier. On the other hand, introducing an asymmetrical load injects a substantial source of intra-subject variability into measurements. This variability does not arise from the person’s individual traits but from the conditions in which the gait is performed. Carrying an additional weight in one hand alters both the amplitude and the overall morphology of GRF signals. Modifications of this type may undermine the stability of biometric features and, in consequence, reduce their discriminative strength. For that reason, examining how asymmetrical loading affects gait-based identification systems appears not only reasonable but necessary, particularly if the goal is to approximate conditions resembling everyday, real-world usage scenarios.

### 1.1. Motivation

The presently published literature concerning human gait recognition contains only a small number of studies that consider asymmetric loading (such as carrying a briefcase) among their analyzed covariates, with a majority of those systems based on video signals [19,20]. Unfortunately, when it comes to using GRFs as the measurement signal, however, there exists a gap concerning this issue within the subject-related literature. The aim of this study is to present a method for the recognition of a person based on their gait through the use of GRFs in cases where the subject is asymmetrically loaded.

### 1.2. Contributions

The main contributions of this work are specified below:

- A biometric system using an ensemble classifier that achieves high-quality person recognition based on GRF signals, even when the human movement pattern is disturbed by asymmetric loading, is described.
- Recognition results for selected base classifiers (deep neural networks) are presented.
- Two methods for result aggregation are proposed and tested, with the impact of parameter selection on the obtained recognition performance for one of them discussed.
- The proposed human recognition algorithm is tested on a large dataset collected by the author of this study, constituting one of the most comprehensive databases described in the literature.

### 1.3. Related Works

In recent years, there has been increased interest in the application of human gait recognition as a biometric. The attractiveness of this parameter, particularly in systems using video cameras, stems from the fact that it can be recorded from a distance. Discreet measurements not requiring the active participation of the user are also possible in biometric systems that use GRFs as input signals. Thus, the gait of a person is well suited for use in monitoring systems, especially those requiring continuous authentication [21]. Moreover, gait is difficult to imitate because fundamental biomechanical constraints limit the extent to which people can voluntarily alter their natural movement patterns. What is more, even if a person attempting to mimic someone else’s gait possesses strong acting skills, it is impossible to observe gait dynamics measured using GRFs without appropriate equipment.

Existing works concerning the utilization of GRFs for the recognition of a person indicate the great potential of such signals for proper identification. In the work of [22], GRFs were divided into parts corresponding to phases of human gait, while the use of the Dynamic Time Warping (DTW) algorithm allowed for the determination of distances between signals. Distances between the signals of different people turned out to be greater than distances between the strides of a particular person, and the employment of the k nearest neighbor (kNN) classifier resulted in the attainment of over 97.37% correct identifications on a sample of 200 people. In the work of [23], in turn, the SVM classifier working on GRFs as input signals achieved 99.3% effectiveness in the recognition of 671 people. The advancements of deep learning have also found reflection in the utilization of deep artificial neural networks in human gait recognition based on GRFs.

To achieve the highest possible accuracy in studies related to human gait biometrics, ensemble classifiers are often employed. One example of such work is that of [24], where a fusion of temporal and spatial holistic pressure information obtained from 127 individuals and recorded using a piezoelectric sensor mat was applied. Better results were achieved by combining the decisions of individual classifiers rather than performing feature-level fusion before classification by a single classifier. In turn, ref. [25] used a multi-stage ensemble of classifiers in which individual classifiers were trained on varying feature vectors for footstep patterns recorded using a special sensor material covering an area of 100 m^2^. In [26], various feature vectors calculated from the three components of the GRF, as well as different base classifiers, were tested. The obtained result of the heterogeneous ensemble achieved an accuracy of 99.65% on a dataset of 322 individuals, demonstrating the effectiveness of this approach.

The issue of the influence of covariates on the performance quality of biometric systems is also addressed in several studies related to human gait recognition [27,28,29]. As previously mentioned, when it comes to GRF-based systems, additional loads carried by the subject are of particular importance. This is because a force plate records force values that are directly dependent on body mass, and body mass itself can be treated as a “weak biometric”. Moreover, one of the advantages of human gait recognition is the possibility of identifying individuals without their conscious participation. Therefore, from the research perspective, an everyday activity such as walking while carrying a briefcase (asymmetric loading) is particularly attractive. Compared to normal walking, this activity constitutes a significant disturbance to the natural gait pattern [30]. In biometric systems, regardless of the type of physical quantities being measured, carrying a briefcase has been shown to significantly degrade the performance of gait-based recognition systems [31]. External asymmetrical loading not only increases the total mass of the body–load system, which naturally leads to higher maximums in GRFs, but also, the distribution of forces between the limbs becomes asymmetrical, and both the shape and timing of foot–ground contact forces begin to shift. In [32], the authors reported that unilateral load carriage increases the gait asymmetry in medial/lateral GRFs and vertical moment GRFs in healthy adults carrying loads corresponding to 10–20% of their body mass in one hand. These changes are accompanied by a subtle lateral lean of the trunk and adjustments of step patterns as the body compensates for the displaced centre of mass. Large-scale population studies point in the same direction. Even a relatively small unilateral load (3.5 kg, for instance) has been shown to noticeably amplify asymmetry in peak GRFs between the left and right foot when compared with unloaded walking conditions [33]. From a biomechanical point of view, these adaptations extend beyond a simple increase in force magnitude. These involve alterations in the trunk posture, step width, and timing of gait phases, thereby changing GRF patterns. Another layer appears at the muscular level. Asymmetrical loading requires increased stabilizing effort from muscles responsible for trunk and lower-limb stabilization. Research that tracks both ground contact forces and muscle activation patterns confirms this tendency, especially under loading conditions with a pronounced asymmetrical component [34].

Lv et al. [35] demonstrated that asymmetrical load carrying reduces gait symmetry and that walking with a load carried in the right hand is characterized by greater gait symmetry than doing it with a load carried in the left. As the load increases, this symmetry decreases proportionally. In [36], the influence of different ways of carrying school backpacks by children on GRFs and temporal characteristics was investigated. Considered carrying techniques included walking without a backpack and with a backpack positioned low on the back, high on the back, or carried by the handle. The results indicated that children took shorter steps and walked at a lower speed, and their vertical GRF components were increased while carrying a backpack compared to walking without one (*p* < 0.01). It was additionally found that, compared to typical gait biomechanics, the greatest changes were observed when the backpack was carried by the handle. The studies of [37] showed how walking with an asymmetric load affects movement patterns and analyzed the individual gait parameters that are subject to change.

The problem of the impact of loading on human gait recognition based on GRFs was most systematically analyzed in the study by Moustakidis [38]. The conducted study involved 40 participants, with two levels of loading (5% and 10% of body weight) and four ways of carrying the load (left hand, right hand, both hands, and on the back). Classification was performed using a kernel-based support vector machine with genetic tuning, and the measurement data were described using signal energy of coefficients obtained from wavelet packet decomposition, with only the most discriminative time–frequency subbands retained after feature selection. In cases where the training set included unloaded gait at a self-selected velocity, and testing was performed on data with asymmetric loading gait, recognition accuracy was 82.7% for loads carried in the left hand and 87.7% for loads carried in the right. In the case of symmetric loading, the recognition rate increased to 90%.

The aim of this study is the presentation of a method for the recognition of a person who is asymmetrically carrying a load based on their gait using GRFs and an ensemble of base classifiers comprising several types of deep neural networks.

## 2. Materials and Methods

### 2.1. Measured Data

**Figure 1:**
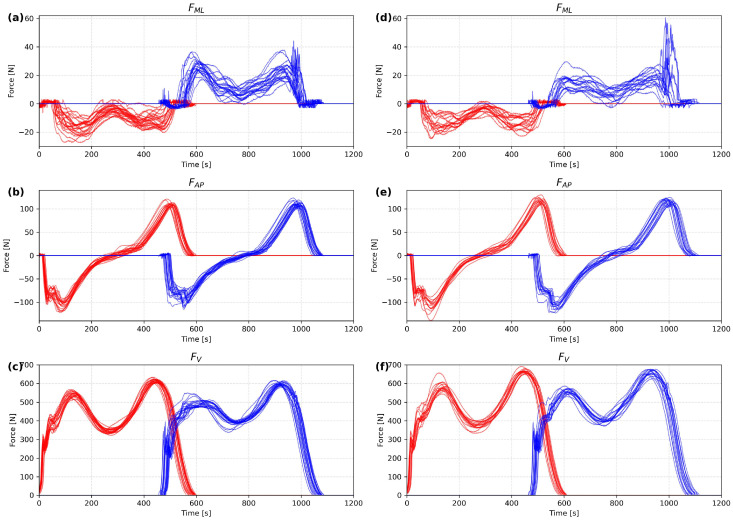
Components of GRF in (**a**) medial/lateral, *F_M__L_*; anterior/posterior, *F_AP_*; and vertical, *F_V_*, directions of the left lower limb (blue line) and of the right one (red line), in sport shoes, without carrying bag—respectively, (**a**), (**b**) and (**c**)—and carrying bag—respectively, (**d**), (**e**) and (**f**). The graph shows a dozen steps of a woman aged 22 years with a body weight of 52.9 kg and height of 159 cm.

Ground reaction force is the force generated during human walking or running between the foot and the ground. It is possible to measure it using force plates made by the Kistler Company (Winterthur, Switzerland), which employ four piezoelectric sensors located at the corners of the platform. The signal measured by the force plates consists of three GRF components: anterior–posterior, *F_AP_*; vertical, *F_V_*; and medial–lateral, *F_ML_* (Figure 1).

Measurements made as part of this study were performed using two Kistler platforms with dimensions of 60 cm × 40 cm, registering data at a frequency of 960 Hz. Ground reaction forces recorded using force plates made by the Kistler Company form a time series, *x*[*n*] = *x*_1_, *x*_2_, *…, x_N_*, where *N* is the number of samples. In general, the duration of the stance phase of human gait depends on a variety of factors and varies; *N* is, therefore, also variable. To facilitate the comparison of two different gait cycles, the length of the longest gait cycle was taken as a reference, and all shorter cycles were padded with zeros. As a result, a dataset with a uniform number of samples was obtained, where *N* = 1471 (which, at the applied sampling frequency of 960 Hz, corresponds to a duration of approximately 1471/960 ≈ 1.53 s). The resulting data vectors were then used in this study without applying normalization to the GRF signals. This decision was made by the intended application scenario for the considered biometrics system. In realistic conditions, the only assumption is that the subject walks across the force plates without any additional acquisition of body mass or other anthropometric parameters. Consequently, normalizing GRF signals with respect to body weight would not be possible under real-world conditions and would reduce the practical usability of the proposed approach.

### 2.2. Augmentation

Data augmentation is commonly utilized in deep learning in order to artificially increase the size and diversity of training sets, which helps in limiting overfitting and improving model generalization, especially when the original dataset is limited with respect to size or diversity. Another expected result with respect to biometrics should be the improvement of classification performance. The current work utilized augmentation methods described below. A more detailed description of these methods is presented in [39,40], while their employment for the classification of human gait has been demonstrated in [41].

#### 2.2.1. Band-Limited Noise Injection

Jittering involves the addition of random low-amplitude noise to a signal, *x*[*n*], in order to increase the resistance of the model to natural measurement fluctuations. In the analyzed code, the noise is additionally low-pass-filtered (4th-order Butterworth filter with a 30 Hz cutoff frequency), limiting its spectrum to a physiologically realistic range.

GRF signals recorded during walking are characterized by predominantly low-frequency content. In many biomechanical studies, the raw GRF signal is subjected to low-pass filtering at cutoff frequencies of approximately 10–20 Hz in order to remove measurement noise and obtain a smoother waveform [42,43]. However, the current augmentation technique filters only the injected noise, not the original GRF signal. Thus, a slightly higher cutoff frequency (30 Hz) was chosen. With this choice, the perturbation stays within a physiologically plausible frequency range of the measured GRF signal while avoiding excessive smoothing that could artificially modify the original waveform dynamics.

From the measurement perspective, this value is also reasonable considering the sampling frequency of the force platform used in this study (960 Hz). The selected cutoff corresponds to only about 3% of the Nyquist frequency (480 Hz), meaning that the perturbation remains strongly limited to the low-frequency part of the spectrum. As a result, the added noise represents small measurement-like fluctuations rather than unrealistic high-frequency distortions of the signal.

Applying this augmentation exclusively during the stance phase is biomechanically justified since this phase generates significant gait dynamic information. During the swing phase of the leg, the signal was previously zeroed. The duration of the stance phase was detected on the basis of the vertical component, *F_V_*, of the GRF. The value of coefficient *a* applied to *F_V_* was half of that applied to the other components.

(1) $$x^{\prime }[n]=x[n]+a\cdot RMS(x)\cdot \tilde{s}[n],$$

where:

- $\tilde{s}(n)$—low-pass filtered white noise; - *RMS*(*x*)—root mean square of an analyzed component of GRF; - *a*—scaling factor; *a*∈{0.01, 0.02}; *a* = 0.01 only for *F_V_*.

#### 2.2.2. Time Shift

Time shifting involves the shifting of the signal along the time axis without changing its shape. In this case, the shift is applied asynchronously to both legs, simulating variability in the synchronization of the lower limbs. This type of augmentation is particularly important in gait recognition, where phase relations between limbs can vary due to, for example, changes in speed or loading. In this study, a shift within the range of ±15 samples was applied. With a sampling frequency of 960 Hz, this shift corresponds to approximately ±15.6 ms. In typical walking, the stance phase takes about 60% of the gait cycle, which is about 0.7 s for a typical stride [42]. Therefore, the applied shift represents less than 2.5% of the stance phase duration. Such a small temporal offset does not alter the overall biomechanical structure of the GRF waveform. Instead, it introduces minor variations in the timing of gait events that naturally occur between successive steps during repeated walking trials.

#### 2.2.3. Random Stance Window Cropping with Resampling

Window cropping is a classic augmentation method used for time series. It involves randomly shortening signal fragments along the time axis and then readjusting the length through resampling. In this case, the operation is limited to the stance phase, preserving the relative structure of the gait cycle while introducing temporal variability. Cropping was applied exclusively to the left leg and did not exceed 3% of the duration of the stance phase. Such a small modification does not alter the overall structure of the GRF waveform but introduces slight variations in its temporal characteristics. This augmentation was applied only to signals corresponding to the left lower limb, allowing for the avoidance of additional variability caused by potential gait asymmetry.

#### 2.2.4. Same-Class Mixup

Mixup involves the creation of new samples as a linear combination of two existing observations. The restriction applied in this study to time series within the same class is particularly important in gait biometrics. For two signals, *x*_1_[*n*], *x*_2_[*n*], recorded from the same individual (signals of the same class), a new sample, *x’*[*n*], was generated using the following formula:

(2) $$x^{\prime }[n]=\gamma x_{1}[n]+(1-\gamma )x_{2}[n],$$

where γ is the mixing coefficient.

The mixing coefficient γ~U(0.85, 0.95) ensures that the augmented signal remains strongly dominated by a single gait instance while introducing controlled intra-class variability. This approach allows for slight variations in the waveform while preserving identity-specific characteristics in GRF-based gait biometrics.

### 2.3. Base Classifiers

Base classifiers play a crucial role in the process of designing ensemble classifiers. In the presented solution, a decision was made to utilize several well-known deep neural networks. In all considered network architectures, the inputs and outputs have the same structure, which allowed for their classification performance to be compared directly. Each network received six components of the GRF profile over the duration of the gait cycle. The temporal dimension of the signals was unified across all samples by applying zero-padding. The output layer of every neural network was configured for the person identification task and contained 215 neurons corresponding to the number of recognition classes, which is the number of individuals included in the dataset.

In all cases, the optimizer used was Adam [44], and the loss function was categorical cross-entropy. Accuracy was adopted as the primary evaluation indicator. Hyperparameters were optimized using Optuna (Version 4.6.0) with early stopping via a Median Pruner [45]. To avoid data leakage, hyperparameter tuning was performed on only 80% of the training data, while the remaining 20% was used to validate the resulting model. During the testing phase, model training with the optimized parameters occurred on the entire training set, while the evaluation was conducted on the test set. Importantly, the separation between the training and testing sets was strictly enforced at the trial level, meaning that individual gait recordings were never shared between these sets. As a result, the model was always evaluated on measurements that had not been seen during the training or validation stages. Additionally, the augmentation procedures described in this work were applied exclusively to the training set, while the test data remained unchanged. This ensured that the reported performance always reflects the model’s ability to generalize to previously unseen measurements rather than memorizing augmented variants of the same signals.

#### 2.3.1. Convolutional Neural Networks

One-dimensional convolutional neural networks (1D-CNNs) are widely used for time series classification because they enable automatic extraction of local temporal patterns without the need for manual feature engineering. The architecture of such networks consists of a sequence of convolutional layers combined with nonlinear functions, often complemented by batch normalization. Depending on the specific implementation, layers reducing temporal resolution, such as pooling layers, may also be employed. The extraction part of the network is typically concluded by global average pooling and a flattening operation, after which a classification layer is applied. The final classification decision is usually realized by one or more fully connected layers or directly by a softmax layer [46].

#### 2.3.2. Hybrid Architecture CNN-LSTM-FC

Hybrid CNN–LSTM–FC architectures combine the ability of convolutional layers to extract local temporal features with the capabilities of LSTM networks to model long-range sequential dependencies. In a typical configuration, the CNN acts as a feature extractor that, together with pooling layers, also reduces the size of the processed sequences, thereby decreasing the computational complexity of the model. The extracted feature maps are then processed by LSTM layers to capture the temporal dynamics of the signal. The final classification decision is realized by a fully connected layer. Karim et al. demonstrated that such a combination can lead to the improvement of classification performance compared to architectures based only on CNN in time series classification tasks [47]. Architectures of this type have also been utilized for the analysis of biomechanical signals [48], including GRFs [49], where simultaneous modeling of both local and sequential temporal dependencies is important.

#### 2.3.3. Residual Neural Networks (ResNets)

ResNet1D is an adaptation of the residual learning concept to one-dimensional time series signals. Skip connections enabling effective training of deep networks by reducing the vanishing gradient problem are a key element of this architecture. In time series applications, ResNet1D employs one-dimensional convolutional blocks, often concluded by global average pooling. Residual architectures have demonstrated high effectiveness in time series classification tasks and are frequently used as strong reference models. Wang et al. have shown that ResNet-type networks achieve competitive performance on standard time series classification benchmarks [50]. For this reason, ResNet1D is currently one of the most used base architectures in this field.

#### 2.3.4. ConvMixer

ConvMixer is an architecture that is based exclusively on convolutional operations and was originally designed for processing image data in the form of patches. The model separates spatial and channel mixing through the use of depthwise and pointwise convolutions. This approach simplifies architecture while maintaining high representational capacity. The authors of [51] demonstrated that ConvMixer achieves competitive results compared to attention-based architectures in computer vision tasks with significantly lower computational complexity. This architecture, after appropriate adaptation, can also be applied to one-dimensional data, such as time series [52].

#### 2.3.5. InceptionTime

InceptionTime is a deep architecture intended for the classification of time series, based on Inception modules adapted to one-dimensional data. Every module, using filters of different sizes, processes the signal in parallel, enabling the simultaneous modeling of temporal dependencies at multiple scales. The architecture employs residual connections and global average pooling, which improves training stability. Fawaz et al. have demonstrated that InceptionTime, particularly in an ensemble configuration, achieves some of the best results on UCR/UEA benchmarks [53]. This model is currently regarded as one of the most effective standard approaches in time series classification.

### 2.4. Ensemble Classifiers

**Figure 2:**
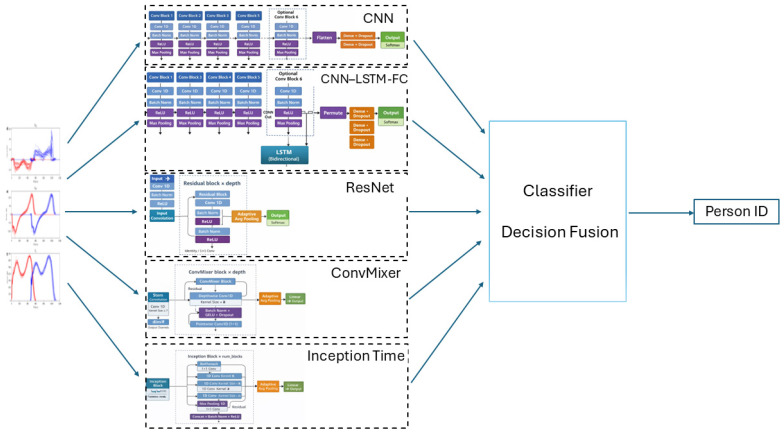
Flowchart of the proposed method.

Recognition of people comes down to the problem of classification, where the number of classes is equal to the sum of the people within the database. For each input vector, *m* (*m* = 1, …, *M*), every one of the *K* base classifiers outputs a vector of posterior probabilities over all classes, i∈C, where *C* denotes the complete set of possible classes. These probabilities are sorted in descending order to obtain a ranking of classes, and the first *T* positions form the *Top-T* list for a given classifier and input sample. Each classifier, therefore, produces an ordered *Top-T* ranking reflecting its confidence in the most likely class assignments. These individual rankings are subsequently aggregated to form the final decision of the classifier ensemble (Figure 2). The current work has made use of two techniques for the fusion of classifier decisions:

- Weighted vote with weight based on rank order;
- Rank-based additive weighted *Top-T* ensemble voting.

Let us define the decision variable:

(3) $$d_{j,p,i}^{\left(m\right)}=\left\{\begin{array}{cc} 1, & \;\mathrm{if}\;\mathrm{classifier}\;j\;\mathrm{ranked}\;\mathrm{class}\;i\;\mathrm{at}\;\mathrm{position}\;p,\\ 0, & \mathrm{otherwise} \end{array}\right.$$

where *j* = 1, …, *K*; *p* = 1, …, *T*.

Here, *p* denotes the position of class *i* within the truncated *Top-T* ranking produced by classifier *j* for sample *m*; classes outside the *Top-T* list are not considered in the definition of $d_{\left(j,p,i\right)}^{\left(m\right)}$.

For every classifier *j* and position *p*∈ {1, *…*, *T*}, and a *Top-p* accuracy is calculated:

(4) $${Acc}_{j,p}=\frac{1}{M}\sum_{m=1}^{M}\sum_{i\in C}I(y_{m}=i)\cdot \sum_{q=1}^{p}d_{j,q,i}^{\left(m\right)},$$

where: 

- *y_m_*ϵ C is the true class label for input vector *m*; - *I*($y_{m}=i$) is an indicator function that assumes a value of 1 when *i* is a true class label of sample *m*, or 0 otherwise.

For every position *p*, classifiers are sorted in descending order according to *Acc_j,p_*. The rank of classifier *j* for position *p* is defined as

(5) $$R_{j,p}=1+\#\left\{l:{Acc}_{l,p}>{Acc}_{j,p}\right\}.$$

In the case of a tie, or when

(6) $${Acc}_{j,p}={Acc}_{l,p},$$

ranking order is determined based on the *Top-1* accuracy:

(7) $${Acc}_{j,1}>{Acc}_{l,1}\;\;\Rightarrow \;\;R_{j,p}<R_{l,p}.$$

If a tie persists after applying Equation (7), the classifiers are assigned the same rank *R_j,p_*.

Based on the rank *R_j,p_* classifier, the weight quality is determined:

(8) $$w_{j,p}=\frac{K+1-R_{j,p}}{K},w_{j,p}\in \left(0,\;1\right].$$

Within the weighted vote strategy, with weight based on rank order, every classifier votes once for one class. At that time, the decision of classifier set $\hat{c}$ is defined as

(9) $$\hat{c}=arg\;\underset{i\in C}{max}\sum_{j=1}^{K}w_{j,1}\cdot d_{j,1,i}^{\left(m\right)}.$$

When it comes to rank-based additive weighted *Top-T* ensemble voting, it is assumed that each classifier casts *T* votes, one for every position *p* obtained in the *Top-T*. The weight assigned to each position was defined analogously to that in Equation (8):

(10) $$f_{p}=\frac{T+1-p}{T},$$

where *p* = 1, …, *T*

Decision $\hat{c}$ of the classifier ensemble, in this case, has been defined as

(11) $$\hat{c}=arg\;\underset{i\in C}{max}\sum_{j=1}^{K}\sum_{p=1}^{T}(\lambda f_{p}+(1-\lambda )\;w_{j,p})\cdot d_{j,p,i}^{\left(m\right)},$$

where λ is the parameter regulating the relative influence of the ranking position, *f_p_*, and the classifier rank-based correction, *w_j,p_*. For 0≤λ≤1, Equation (11) corresponds to a standard weighted combination of both components, whereas values of λ>1 increase the influence of the ranking position relative to the classifier quality term.

The decision rules defined in Equations (9) and (11) may occasionally produce identical maximal scores for more than one class. In such situations, the tie is resolved by selecting the class indicated at the *Top-1* position. If the *Top-1* indication is also non-unique, the ensemble output is set to *None*, meaning that no class is assigned.

The present work utilized *K* = 5 base classifiers, and with the use of rank-based additive weighted *Top-T* ensemble voting, each one employed *Top-T* = 5.

### 2.5. The Study Group

The study was conducted in the laboratory of the Faculty of Mechanical Engineering at the Bialystok University of Technology on a group of 215 healthy individuals, including 92 women and 123 men. Study subjects were 21.34 ± 1.16 years old, with a body mass of 74.33 ± 16.63 kg and a height of 174.41 ± 9.49 cm. All participants were informed about the purpose and manner of testing and signed appropriate consent forms. They were allowed to withdraw from the study at any stage without providing a reason. During the trial, upon a signal from the researcher, the participants walked along a longer than 10 m measuring path in which two Kistler force plates were hidden. Participants were not informed about the presence or location of the force plates. In cases where a participant missed the plate or stepped on its edge, the measurement was repeated with a slight adjustment to the trial starting point. Each participant walked in their own sports footwear and at their own pace. To avoid the impact of fatigue on gait, a short 1–2 min break was taken after every 10 passes. The test was conducted in two stages. Normal gait was recorded during the first stage, and in the second stage, participants were asked to walk while carrying a briefcase weighing 4.6 kg. The weight of the briefcase corresponded to that of a computer bag containing a laptop and a few documents. Each participant could carry the briefcase in the hand of their own choosing, but they were not allowed to change hands during the measurements. Each stage of the testing (walking without and with the briefcase) was repeated multiple times (14–20 trials), resulting in the recording of a total of 7351 gait cycles, of which 3960 represented typical gait (without briefcase) and 3391 strides with the briefcase. The study was conducted in accordance with ethical standards. All participants provided informed consent, and the protocol was approved by the Bioethics Commission of the Regional Medical Chamber in Bialystok (Poland) and the Bioethics Commission of the Medical University of Bialystok.

## 3. Results and Discussion

The study assumed three testing scenarios:

(a) Scenario A—Referential—Both the training and the testing set contained only data describing test subjects’ gait without the briefcase. In this scenario, the evaluation was performed using 10-fold cross-validation. Consequently, no measurement used during training in a given iteration appeared in the corresponding test subset, and the classifier was always evaluated on trials that were not used during model training.
(b) Scenario B—Realistic—The training set consisted only of data characterizing the gait of participants without the briefcase, while the testing set consisted only of data recorded while carrying the briefcase. This scenario reflects a realistic deployment condition, where the system is trained under standard conditions but evaluated under a covariate affecting gait dynamics.
(c) Scenario C—The data were the same as in scenario B, but with an augmentation of data from the training set. To realize this goal, eight additional time series were obtained using techniques (and combinations of them) presented in Section 2.2. These included “jittering”, “time shifting”, “window cropping”, “jittering + time shifting”, “jittering + window cropping”, “time shifting + window cropping”, “jittering + mixup”, “jittering + window shifting + window cropping”. It should be noted that the evaluation protocol ensured that the classifier was always tested on original gait data that were not used during training, which reduces the risk of overfitting and limits the possibility that the model exploits artifacts related to individual trials rather than subject-specific gait characteristics.

In all scenarios, the training and testing sets were exclusive and included data from all individuals.

The parameter ranges for individual network components explored using the Optuna framework are summarized in Table 1, Table 2, Table 3, Table 4 and Table 5. The search space was defined based on prior experience while taking into account hardware limitations and computational complexity. All experiments were conducted on a workstation equipped with an AMD Ryzen 9 7900 processor, with an RTX 5070 Ti GPU and 64 GB of RAM.

In the case of CNN models, each convolutional block applies a one-dimensional convolution followed by batch normalization, ReLU activation, and max-pooling. An additional sixth convolutional block was optionally included in selected optimization trials.

Each convolutional block in the CNN-LSTM-FC model applies a one-dimensional convolution followed by batch normalization, ReLU activation, and max-pooling. The resulting feature sequences are then processed by an LSTM layer to capture temporal dependencies.

In the case of ResNet, each residual block is composed of two one-dimensional convolutional layers, each followed by batch normalization and ReLU nonlinearity, with an identity shortcut used to preserve the input information. Global feature aggregation is achieved through adaptive average pooling.

Each ConvMixer block applies a depthwise one-dimensional convolution and a subsequent pointwise convolution, combined with batch normalization and GELU nonlinearity. Dropout and residual connections are used to improve generalization, while global feature representations are obtained through adaptive average pooling.

Each Inception block applies several one-dimensional convolutional branches with different kernel sizes, preceded by a bottleneck convolution and integrated through a residual connection. Global feature representations are obtained using adaptive average pooling.

It should be emphasized that during training in Scenario A, a 10-fold cross-validation was applied. Since training and test data were collected under different conditions in Scenarios B and C, applying cross-validation did not make sense. The exact numbers for both training and testing sets have been presented in Table 6.

Given the adopted assumptions, a total of 60 different artificial deep neural network models were obtained. For this reason, their detailed architectures are not reported.

### 3.1. Recognition Using Base Classifiers

The *Top-1* and *Top-5* training results for deep neural networks presented in Section 2.3 are described in Table 7, Table 8 and Table 9. Accuracy values are reported in percentage form.

The results obtained for all scenarios were good or very good. In the case of Scenario A, all networks achieved similar accuracy (*Top-1*: 98.43–98.91%). These values are much higher than those presented in [46], where CNN and GRF networks were able to achieve a value of 96.57%. The difference is caused by a sample larger than that of this study (322 people), as well as a lack of optimization of the neural network architecture. In the work of [23], classification accuracy for a similar task was reported for a study group of 671 participants, with the CNN classifier achieving 95.8% correct classifications, while the SVM classifier reached an accuracy of 99.3%, which significantly exceeds the result presented within this work.

With respect to Scenario B, a significant decrease (ranging from 3.07 to 5.12 percentage points) in accuracy (*Top-1*) can be observed compared to the results obtained in Scenario A. The smallest drop in accuracy was recorded for the InceptionTime network, suggesting that the simultaneous detection of local temporal patterns and longer-term dependencies allows for the capture of specific gait characteristics of an individual despite the disturbances applied in this study. A decline in recognition quality clearly indicates a significant impact caused by changes in GRFs, both in terms of their absolute values and the relationships between them, generated by a person carrying a briefcase compared to the same person walking without one. It should be emphasized that applying neural network structure optimization to the training set intensified problems in the recognition of people on the basis of data from the test set. Nevertheless, these results are significantly better than those previously reported in the literature. In the study by [38], research conducted for a study group of 40 people under similar conditions achieved a maximum recognition accuracy of only 87.7%. This result was obtained for loading conditions corresponding to 5% or 10% of body mass.

The application of data augmentation (Scenario C) resulted in a systematic improvement in Top-1 accuracy for all analyzed architectures. The largest increases were observed for the classic CNN (from 94.34% to 97.99%) and ConvMixer (from 93.31% to 95.81%), suggesting that augmentation is particularly beneficial for models with a lower capacity for generalization when training samples are limited. In the case of the InceptionTime architecture, Top-1 accuracy increased to 97.02%, confirming that even models with high baseline effectiveness can further benefit from the introduction of controlled temporal variance during the training phase. These results support the thesis presented by [39,40], among others, that augmentation improves the performance of deep neural networks for time series data, including GRFs.

In nearly all cases, *Top-5* accuracy exceeds 99%. From the perspective of biometric applications, these *Top-5* results confirm that even in instances of incorrect *Top-1* classification, the correct individual is almost always present among the several most likely candidates. This is particularly significant in identification scenarios using ensemble classifiers, including in systems employing multimodal fusion, where the candidate list generated based on GRFs can be further narrowed down using additional information sources.

**Figure 3:**
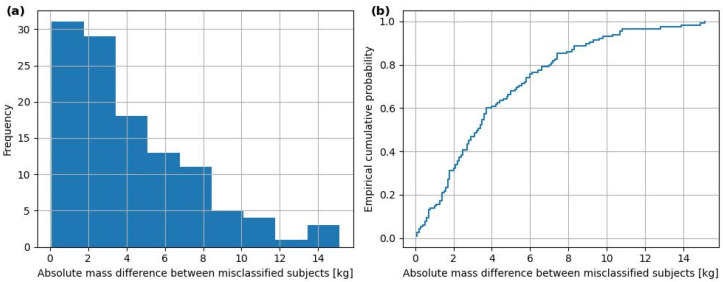
Distribution of absolute body mass differences between pairs of misclassified subjects for ConvMix network in Scenario B: (**a**) Frequency histogram. (**b**) Empirical cumulative distribution function (ECDF).

Figure 3 presents a histogram of the number of erroneous recognitions relative to the absolute difference in body mass between misidentified individuals for the ConvMix network. The histogram exhibits a strongly right-skewed distribution, which means that the majority of misclassifications occurred between individuals with very similar body masses. Specifically, the highest error frequency is observed for mass differences below 3 kg. As the absolute mass difference increases, the number of misclassifications decreases rapidly, and errors involving individuals with a body mass difference of more than 10 kg occur only sporadically. These findings are confirmed by the empirical cumulative distribution function presented in Figure 3b. Approximately 65% of all incorrect recognitions involve pairs of individuals whose mass difference does not exceed 4.6 kg, which corresponds to the mass of the carried briefcase. Over 95% of the observed misclassifications occur between individuals whose body mass differs by less than approximately 11–12 kg. Only a marginal fraction of errors is associated with larger differences in mass. The obtained results indicate that the proposed model primarily confuses individuals with similar body mass, while individuals significantly differing in mass are typically correctly distinguished. Such behavior suggests that the classification errors are not random but follow an ordered pattern arising from physiologically significant similarities between individuals. In the context of biometric gait recognition, this is consistent with the expectation that body mass influences gait dynamics and, consequently, GRFs utilized by the model.

### 3.2. Recognition Using Ensemble Classifiers

**Figure 4:**
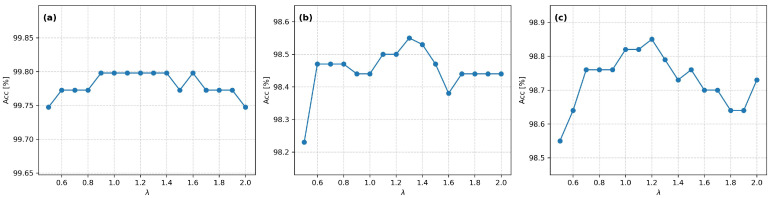
Classification accuracy depending on the λ parameter for test scenarios: (**a**) Scenario A; (**b**) Scenario B; (**c**) Scenario C.

The results for the recognition of people performed by ensemble classifiers, depending on the strategy of vote combination of base classifiers, are presented in Table 10. Since the parameter *λ* in Formula (11) can assume various values, a series of experiments was conducted to verify the classification quality of the ensemble, where *λ*ϵ(0; 5>, Δ*λ* = 0.1. Therefore, the best obtained classification results for the rank-based additive weighted *Top-T* ensemble voting strategy are presented in Table 10. For Scenario A, the best results were achieved for λ ϵ < 0.9; 1.4 > Δ*λ* = 0.1; for Scenario B, *λ* = 1.3; and for Scenario C, it was *λ* = 1.2 (Figure 4).

For all analyzed scenarios, the use of ensemble classifiers leads to an improvement in recognition accuracy compared to recognition based on a single classifier. The largest increase in accuracy, by 2.78 percentage points (from 95.77% to 98.55%), occurred for Scenario B. Significantly smaller increases were observed for the remaining scenarios, amounting to 0.86 percentage points for Scenario C and 0.89 percentage points for Scenario A. Despite the relatively small absolute values, this quality improvement is significant since it brings the result closer to 100% of correct recognition. Regardless of the scenario, the rank-based additive weighted *Top-T* ensemble voting strategy achieves better results than the weighted vote with a weight based on rank order. The difference between these strategies ranges from 0.08 to 0.59 percentage points for optimal *λ* values.

An analysis of the influence of parameter λ on the obtained values shows that there is a relatively wide range over which the impact of the λ value is slight. However, the optimal value of *λ*, located slightly above unity, regardless of the scenario, indicates that the best results are achieved by moderately privileging *Top-T* information over the global quality of individual classifiers. The obtained results confirm the validity of using ensemble classifiers as an effective and robust solution for practical biometric gait recognition systems.

### 3.3. Ablation Study

To examine the contribution of individual classifiers to ensemble behavior, a leave-one-out ablation analysis was carried out. In each variant, one model was removed, while the remaining components of the system were left unchanged. The results obtained for the three experimental scenarios are summarized in Table 11 and Table 12. All reported ties were obtained according to the voting procedures defined in Equations (9) and (11).

In Scenario A, the complete ensemble achieved an accuracy of 99.72 ±0.22%. Removing any single classifier produced only marginal changes, and the largest decrease did not exceed 0.13 percentage points. This drop appeared when either ResNet or InceptionTime was excluded, which suggests that both architectures capture patterns not fully represented by the remaining models. An additional detail becomes visible in the voting stage: ties occur only after removing any of the classifiers, whereas the full set of classifiers produces none.

The situation in Scenario B is less uniform. The full set of classifiers reached 97.94% accuracy, yet removing some models led to noticeably larger changes. The strongest effect followed the exclusion of ResNet and CNN, reducing performance by 0.83 and 0.80 percentage points, respectively. These configurations also produced a larger number of ties, with 41 cases observed when ResNet was removed. The pattern is difficult not to notice, as it is associated with larger performance drops and is typically accompanied by a higher number of voting ties, indicating that an even number of classifiers play an important role in their formation.

Scenario C reveals a slightly different structure. The full set of classifiers obtained 98.47% accuracy. Removing CNN resulted in the largest decrease (0.65 percentage points), while excluding ConvMixer or InceptionTime slightly improved accuracy. Even in those cases, however, the number of ties increased noticeably. This suggests that classifiers whose removal does not necessarily reduce accuracy may still help maintain a more coherent ranking of candidate classes.

It should be noted that the ties reported in Table 11 occur exclusively during the voting stage. Whenever identical scores appear, the ambiguity is resolved using the prediction of the best base classifier, ensuring that the ensemble always generates a single final label.

Table 12 presents the corresponding ablation results obtained with the rank-based additive weighted *Top-T* ensemble voting scheme. In Scenario A, the full ensemble achieved 99.80 ± 0.19% accuracy. Removing any individual classifier again had only a minor impact. The largest decrease occurred after excluding CNN (0.05 percentage points), whereas removing ResNet slightly improved accuracy. The number of ties remained extremely low, typically between zero and two cases.

In Scenario B, the full set of classifiers reached 99.55 of correct classifications. The largest decline followed the removal of ResNet (0.47 percentage points), while excluding CNN or CNN + LSTM_FC produced smaller reductions. At the same time, the number of ties increased substantially, reaching 27 when ResNet was absent. It should be underlined that in the case of 27 ties, the decision based on the *Top-1* of the best classifier affects almost 0.8% of all recognition. Interestingly, removing InceptionTime slightly improved accuracy to 98.58%, although this configuration still generated several ties.

Scenario C follows a somewhat different pattern. The full set of classifiers achieved 98.85% accuracy. Removing CNN produced the largest decrease (0.56 percentage points), and excluding CNN+LSTM+FC also reduced performance. The remaining architectures had only a modest influence on accuracy, yet their removal often increased the number of ties, particularly in the case of ResNet.

Another aspect visible in Table 12 is the values of the parameter λ, which gives the best results. Its optimal value depends on the composition of the ensemble. When all classifiers are present, larger values of λ are typically preferred, whereas removing certain models occasionally shifts the optimum toward smaller values. This indicates that the aggregation mechanism adapts to both the number and diversity of the participating classifiers.

When both ablation analyses are considered together, a coherent picture begins to emerge. Although the two experiments rely on different aggregation strategies, the overall behavior of the ensemble remains remarkably stable. The same classifiers repeatedly appear as the most influential components of the system, and their removal tends to produce the largest performance changes. In particular, CNN and ResNet frequently stand out as architectures whose absence most strongly affects the ensemble.

A second recurring observation concerns the relationship between accuracy and the number of ties. The configuration that produces larger performance drops typically generates more ties during the voting stage. Certainly, an even number of classifiers in an ensemble makes it easier to achieve a tie. In addition, the rule for breaking ties uses a simple algorithm that abandons the set of classifiers and relies on the decision of only one of them.

Taken together, these results suggest that the effectiveness of the ensemble stems primarily from the interaction between several diverse architectures rather than from the dominance of any single model.

### 3.4. Computational Latency of the Ensemble Pipeline and Its Components

In biometric recognition systems, the time required to produce an identification decision is very important. The latency measurements reported in this section refer exclusively to the operation of the already trained system, i.e., the inference stage. The training itself is far more time-consuming and strongly dependent on hyperparameters selected by the optimization procedure implemented in Optuna, as well as on the size of the training sequence. In the present study, the training stage lasted from several hours in the case of the CNN model to approximately 30 hours for the InceptionTime architecture.

Table 13 reports the inference latency of each of the five base classifiers. To enable reliable timing measurements, the GPU was warmed up prior to the evaluation, and the function torch.cuda.synchronize() was used to prevent asynchronous execution from distorting the results. The benchmark consisted of 100 independent measurements. In every run, a single pattern was processed, the processing time was recorded, and the final statistics were calculated as the mean latency together with its standard deviation.

It is quite easy to notice that the CNN model clearly exhibits the lowest latency, processing a sample in roughly 0.32 ms, which corresponds to more than 3100 samples per second (Table 13). The other architectures of neural networks operate at a noticeably slower pace. ResNet and CNN+LSTM+FC occupy the middle ground, both requiring slightly above 1 ms per sample. ConvMixer introduces a further delay, while InceptionTime emerges as the computationally heaviest model, exceeding 3 ms per inference. Despite these differences, all base classifiers remain within a time scale compatible with real-time operation.

The computational burden introduced by the ensemble fusion is negligible (Table 14). Both voting mechanisms execute in the microsecond range several orders of magnitude faster than the neural network inference itself. The weighted vote with a weight based on rank order is the faster of the two strategies, although even the rank-based additive weighted *Top-T* ensemble voting scheme remains extremely lightweight.

When the complete processing chain is considered (Table 15), the latency is almost entirely determined by the inference of the five neural networks. The ensemble fusion stage contributes only a negligible fraction of the total processing time. Consequently, the full pipeline requires slightly above 7 ms per input sample with nearly identical results for both voting schemes. This corresponds to a throughput slightly exceeding 140 processed samples per second for each voting method, confirming that the ensemble layer does not introduce any practically relevant delay in the identification process.

### 3.5. Discussion

The results presented in the previous subsections were obtained based on GRFs from a study involving a fairly large group of participants. It should be emphasized that the research material covers a relatively homogeneous group of participants, which naturally increases the difficulty level of the classification task. Despite this limitation, the recognition accuracies achieved in this study, reaching 99.8% in the reference scenario involving only natural gait, rank among the top results reported in the literature on biometric gait recognition. A comparable level of effectiveness was presented in the work of [54]. It should be noted, however, that those experiments were conducted on a relatively small group of only 50 individuals, a fact that may significantly inflate reported recognition results. One example of an approach that was able to achieve very high effectiveness is the work of [55], where the proposed Gait-ViT model enabled the correct identification of 99.51% to 100% of gait cycles. Importantly, this effectiveness was achieved on very large datasets, such as the OU-LP database encompassing 3916 participants. At the same time, it should be noted that in both [54,55], gait representation is based on the analysis of video sequences, and therefore, a direct comparison of those results with the results presented in this work is debatable

A study by [19] utilized the CASIA-B covariate dataset, containing video sequences of the gait of 124 individuals, and included cases of bag carrying. A system trained exclusively on gait sequences without a load (CASIASetA) achieved approximately 60% effectiveness for test sequences with a bag (CASIASetB) when using the classic GEI representation. The application of a Joint Sparsity model and classification based on *ℓ1* norm minimization allowed for an increase in the recognition accuracy of individuals carrying a bag to 92.7%. A similar generalization problem is analyzed in [20], where a generative Ab-GAN approach was proposed to remove the influence of carried objects at the level of silhouette representation. In experiments, also conducted using a protocol of training on sequences without a load and testing on sequences with a bag, the authors achieved an average Rank-1 recognition accuracy of 98.3% on the CASIA-B database. This result is similar to the one obtained in the present work, with the previously mentioned stipulation regarding the different signals used for recording human gait.

In the context of studies utilizing foot pressure measurements, the results presented in the present work indicate a clear advantage of the proposed approach over most existing solutions. In the work of [56], a specialized convolutional–recurrent neural network (KineticNet) was employed, which, utilizing the vertical and anterior/posterior GRF components, along with a single center of pressure coordinate, enabled the re-identification of 118 subjects with approximately 96% accuracy. Although this result confirms the utility of GRF signals for identification tasks, it remains notably lower than the results achieved in the present study. In the work of [25], a kNN group of classifiers was employed, achieving results exceeding 95%, but the analysis included only 10 participants. Yun, on the other hand (utilizing UbiFloorII and MLP), reported an error rate of approximately 1%, but in this case, the number of participants was also small [57]. A study by [18], analyzing foot pressure patterns, obtained an error rate ranging from 0.6% to 7.1% for 104 individuals and 520 strides. As previously mentioned, in the work of [23], the application of an SVM classifier yielded an accuracy of 99.3%, but this value remains significantly lower than that achieved in the present study using ensemble classifiers.

The most significant result, however, is the achievement of high-quality classifications in scenarios involving gait disturbances caused by asymmetric loading. Most importantly, the results obtained in this study, 98.55% and 98.85% accuracy, decidedly outperform those achieved in similar works completed earlier (such as the 87.7% accuracy in [38]). Furthermore, in the study of [58], where GRFs were parameterized using seven variables, an accuracy of up to 95.74% was achieved, and that is in a task of detecting whether or not a person was carrying a briefcase. Applying the parameters described in that work to the task of the recognition of a person in a scenario analogous to the one used in this study only yields approximately 77% accurate recognition.

## 4. Conclusions

The research conducted confirms that GRFs constitute a valuable biometric modality for the identification of people based on their gait, including under conditions of asymmetric loading. The employed deep learning architectures achieved high identification effectiveness, with the best results obtained for models capable of multi-scale temporal signal analysis. The introduction of data augmentation led to a systematic improvement in *Top-1* accuracy for all analyzed classifiers while simultaneously maintaining very high *Top-5* values. The analysis of results indicates that classification errors are local in nature and primarily involve individuals with similar body mass, as illustrated in Figure 3, where most misclassifications occur between individuals whose body mass differs only slightly. The results obtained using ensemble classifiers suggest that the rank-based additive weighted *Top-T* ensemble voting strategy enables the achievement of very high identification effectiveness, even in large and relatively homogeneous study populations tested under asymmetric loading. Furthermore, appropriately designed augmentation methods can effectively increase the resistance of GRF-based biometric systems to variability in gait conditions, making them a promising solution for practical applications.

Future studies should test the quality of the proposed solution with carried loads heavier than those considered here, as well as in cases of symmetric loading (such as in the form of a backpack). Additionally, other covariates, such as walking in different footwear—especially walking in high heels—should be examined.

## Figures and Tables

**Table 1:** Hyperparameter search space used for optimization of the CNN model.

Network Part	Hyperparameter	Search Space
Convolutional block 1	Number of filters Kernel size	{16, 32, 64} {3, 5, 7}
Convolutional block 2	Number of filters Kernel size	{32, 64, 128} {3, 5}
Convolutional block 3	Number of filters Kernel size	{64, 128, 256} {3, 5}
Convolutional block 4	Number of filters Kernel size	{128, 256, 512} {3}
Convolutional block 5	Number of filters Kernel size	{256, 512, 1024} {3}
Convolutional block 6 (optional)	Number of filters Kernel size	{256, 512} {3}
Fully connected layer 1	Number of neurons	{512, 1024}
Fully connected layer 2	Number of neurons	{256, 512, 700}
Regularization	Dropout rate (layer 1)	[0.1, 0.6]
	Dropout rate (layer 2)	[0.1, 0.6]
Training parameters	Batch size Learning rate	{16, 32, 64} [10^−5^, 10^−3^] (logarithmic scale)

**Table 2:** Hyperparameter search space used for optimization of the CNN-LSTM-FC model.

Network Part	Hyperparameter	Search Space
Convolutional block 1	Number of filters Kernel size	{16, 32, 64} {3, 5, 7}
Convolutional block 2	Number of filters Kernel size	{32, 64, 128} {3, 5}
Convolutional block 3	Number of filters Kernel size	{64, 128, 256} {3, 5}
Convolutional block 4	Number of filters Kernel size	{128, 256, 512} {3}
Convolutional block 5	Number of filters Kernel size	{256, 512, 1024} {3}
Convolutional block 6	Number of filters Kernel size	{256, 512} {3}
LSTM layer	Hidden state size Number of layers Dropout rate Bidirectional	{64, 128, 192, 256, 320, 384, 448, 512} {1, 2} [0.0, 0.5] {False, True}
Fully connected layer	Number of neurons	{128, 256, 512, 1024}
Regularization	Dropout rate (FC)	[0.1, 0.5]
Training parameters	Batch size Learning rate	{16, 32, 64, 128} [10^−5^, 10^−3^] (logarithmic scale)

**Table 3:** Hyperparameter search space used for optimization of the ResNet model.

Network Part	Hyperparameter	Search Space
Input convolution	Number of filters Kernel size	{16, 32, 64} 7 (fixed)
Residual blocks	Number of residual blocks (depth)	{2, 3, 4, 5, 6}
Training parameters	Batch size Learning rate	{16, 32, 64} [10^−5^, 10^−3^] (logarithmic scale)

**Table 4:** Hyperparameter search space used for optimization of the ConvMixer model.

Network Part	Hyperparameter	Search Space
Stem Convolution	Number of filters Kernel size	{16, 32, 64, 128} 7 (fixed)
ConvMixer blocks	Number of blocks (depth) Kernel size (depthwise conv) Dropout rate	{4, 5, 6, 7, 8} {3, 5, 7, 9} [0.0, 0.4]
Training parameters	Batch size Learning rate	{16, 32, 64, 128} [10^−5^, 10^−3^] (logarithmic scale)

**Table 5:** Hyperparameter search space used for optimization of the InceptionTime model.

Network Part	Hyperparameter	Search Space
Inception blocks	Number of inception blocks	{3, 4, 5, 6, 7, 8, 9}
Inception module	Number of filters per branch Base kernel size Bottleneck channels	{16, 32, 64, 96} {7, 9, 11} {16, 32}
Regularization	Dropout rate	[0.0, 0.5]
Training parameters	Batch size Learning rate	{32, 64, 128} [10^−5^, 10^−3^] (logarithmic scale)

**Table 6:** Number of training and testing sets for individual scenarios.

	Scenario A	Scenario B	Scenario C
Training set	3565 *	3961	35,649
Testing set	396 *	3391	3 391

* For most of the folds.

**Table 7:** Average accuracy (±standard deviation) for *Top-1* and *Top-5* for individual classifiers in Scenario A.

Type of Classifier	Acc *Top-1* (%)	Acc *Top-5* (%)
CNN	98.61 ± 0.87	99.77 ± 0.22
CNN + LSTM + FC	98.66 ± 0.58	99.82 ± 0.17
ResNet	98.91 ± 1.18	99.82 ± 0.27
ConvMixer	98.43 ± 1.13	99.82 ± 0.12
InceptionTime	98.84 ± 0.75	99.92 ± 0.17

**Table 8:** Accuracy for *Top-1* and *Top-5* for individual classifiers in Scenario B.

Type of Classifier	Acc *Top-1* (%)	Acc *Top-5* (%)
CNN	94.34	99.12
CNN + LSTM + FC	94.54	98.73
ResNet	94.96	99.59
ConvMixer	93.31	99.29
InceptionTime	95.77	99.38

**Table 9:** Accuracy for *Top-1* and *Top-5* for individual classifiers in Scenario C.

Type of Classifier	Acc *Top-1* (%)	Acc *Top-5* (%)
CNN	97.99	99.23
CNN + LSTM + FC	96.28	99.50
ResNet	96.93	99.44
ConvMixer	95.81	99.47
InceptionTime	97.02	99.56

**Table 10:** Accuracy for both voting strategies and different scenarios.

	Scenario A	Scenario B	Scenario C
Weighted vote with weight based on rank order	99.72% ± 0.22%	97.94%	98.47%
Rank-based additive weighted *Top-T* ensemble voting	99.80% ± 0.19%	98.55%	98.85%

**Table 11:** Results of the leave-one-out ablation analysis of the ensemble classifiers for weighted vote with a weight based on rank order.

	Model Removed	Accuracy (%)	Δ Accuracy (%)	Ties
Scenario A	None	99.72 ± 0.22	0	0
	CNN	99.67 ± 0.17	−0.05	5
	CNN + LSTM + FC	99.67 ± 0.21	−0.05	5
	ResNet	99.59 ± 0.36	−0.13	7
	ConvMixer	99.69 ± 0.26	−0.03	3
	InceptionTime	99.59 ± 0.24	−0.13	6
Scenario B	None	97.94	0	0
	CNN	97.14	−0.80	32
	CNN + LSTM + FC	97.26	−0.68	28
	ResNet	97.11	−0.83	41
	ConvMixer	97.61	−0.33	25
	InceptionTime	97.79	−0.15	24
Scenario C	None	98.47	0	0
	CNN	97.82	−0.65	11
	CNN + LSTM + FC	98.35	−0.12	15
	ResNet	98.47	0	13
	ConvMixer	98.61	0.14	22
	InceptionTime	98.56	0.09	11

**Table 12:** Results of the leave-one-out ablation analysis of the ensemble classifiers for rank-based additive weighted *Top-T* ensemble voting.

	Model Removed	Accuracy (%)	Δ Accuracy (%)	Value of λ	Ties
Scenario A	None	99.80 ± 0.19	0	0.9–1.4	0
	CNN	99.75 ± 0.21	−0.05	0.8, 0.9, 1.1	1–2
	CNN+LSTM+FC	99.77 ± 0.22	−0.03	1.1–1.2	1
	ResNet	99.82 ± 0.12	0.02	0.8–0.9	0
	ConvMixer	99.80 ± 0.20	0	0.8, 0.9, 1.1–1.4	1
	InceptionTime	99.80 ± 0.24	0	0.9	0
Scenario B	None	98.55	0	1.3	1
	CNN	98.32	−0.23	1	19
	CNN+LSTM+FC	98.29	−0.26	1	18
	ResNet	98.08	−0.47	1	27
	ConvMixer	98.29	−0.26	1.2	8
	InceptionTime	98.58	0.03	1.4	5
Scenario C	None	98.85	0	1.2	2
	CNN	98.29	−0.56	1–1.4	0–16
	CNN+LSTM+FC	98.47	−0.38	0.9, 1, 1.2	3–18
	ResNet	98.76	−0.09	1	19
	ConvMixer	98.82	−0.03	0.8–0.9	2
	InceptionTime	98.73	0.12	1.1	5

**Table 13:** Average inference latency of the individual base classifiers for a single input sample.

Model	Latency Mean (ms)	Latency Std (ms)	Samples per Second
CNN	0.319170	0.114409	3133.13
CNN + LSTM + FC	1.117724	0.095759	894.675
ResNet	1.039989	0.172970	961.549
ConvMixer	1.470009	0.270911	680.268
InceptionTime	3.169560	0.343075	315.501

**Table 14:** Computational latency of the ensemble decision strategies.

Voting Methods	Latency Mean (ms)	Latency Std (ms)	Samples per Second
Weighted vote with weight based on rank order	0.002662	0.001064	375657.3
Rank-based additive Weighted *Top-T* ensemble voting	0.008895	0.001483	112422.7

**Table 15:** Latency breakdown of the complete identification pipeline, including neural inference and ensemble fusion.

Stage	Latency (ms)	
	Weighted Vote with Weight Based on Rank Order	Rank-Based Additive Weighted *Top-T* Ensemble Voting
Neural networks inference (5 models)	7.116452	7.116452
Ensemble fusion	0.002662	0.008895
Total pipeline	7.119114	7.125347

## Data Availability

The dataset is available upon request from the author.

## Abbreviations

The following abbreviations are used in this manuscript:

GRF	Ground Reaction Forces
IMU	Inertial Measurement Unit
DTW	Dynamic Time Warping
kNN	k Nearest Neighbor classifier
SVM	Support Vector Machine
RMS	Root Mean Square
ADAM	Adaptive Moment Estimation
CNN	Convolutional Neural Network
LSTM	Long Short-Term Memory
FC	Fully Connected
ResNet	Residual Neural Network
1D	One Dimensional
ReLU	Rectified Linear Unit
GELU	Gaussian Error Linear Unit
ECDF	Empirical Cumulative Distribution Function
MLP	Multi-Layer Perceptron
